# ABC-tool reinvented: development of a disease-specific ‘Assessment of Burden of Chronic Conditions (ABCC)-tool’ for multiple chronic conditions

**DOI:** 10.1186/s12875-019-1075-8

**Published:** 2020-01-13

**Authors:** Esther A. Boudewijns, Danny Claessens, Onno C. P. van Schayck, Lotte C. E. M. Keijsers, Philippe L. Salomé, Johannes C. C. M. in ‘t Veen, Henk J. G. Bilo, Annerika H. M. Gidding-Slok

**Affiliations:** 1 Department of Family Medicine, Care and Public Health Research Institute (CAPHRI), Maastricht University, P.O. Box 616, 6200 MD Maastricht, the Netherlands; 2 General practitioner, IJsselstein, The Netherlands; 3 Department of Pulmonology, Franciscus Gasthuis & Vlietland, Rotterdam, The Netherlands; 4 Department of Pulmonology, Erasmus MC, Rotterdam, The Netherlands; 5 Department of Internal Medicine, University of Groningen, University Medical Centre Groningen, Groningen, The Netherlands

**Keywords:** Burden of disease, Chronic disease, ABCC-tool, Self-management, Questionnaire, Asthma, Diabetes mellitus type 2, COPD, Shared decision making, Patient-centered care

## Abstract

**Background:**

Numerous instruments have been developed to assess patient reported outcomes; most approaches however focus on a single condition. With the increasing prevalence of multimorbidity, this might no longer be appropriate. Moreover, a more comprehensive approach that facilitates shared decision making and stimulates self-management is most likely more valuable for clinical practice than a questionnaire alone. This study aims to transform the Assessment of Burden of Chronic Obstructive Pulmonary Disease (COPD) (ABC)-tool into the Assessment of Burden of Chronic Conditions (ABCC)-tool for COPD, asthma, and diabetes mellitus type 2 (DM2). The tool consists of a scale, a visualisation of the outcomes, and treatment advice.

**Methods:**

Requirements for the tool were formulated. Questionnaires were developed based on a literature study of existing questionnaires, clinical guidelines, interviews with patients and healthcare providers, and input from an expert group. Cut-off points and treatment advice were determined to display the results and to provide practical recommendations.

**Results:**

The ABCC-scale consists of a generic questionnaire and disease-specific questionnaires, which can be combined into a single individualized questionnaire for each patient. Results are displayed in one balloon chart, and each domain includes practical recommendations.

**Conclusions:**

The ABCC-tool is expected to facilitate conversations between a patient and a healthcare provider, and to help formulate treatment plans and care plans with personalised goals. By facilitating an integrated approach, this instrument can be applied in a variety of circumstances and disease combinations.

## Background

Over the last years, chronic conditions have emerged as a major challenge to global health [1]. Concomitantly, Patient Reported Outcome Measures (PROMs), such as quality of life (QoL) and patients’ experienced burden of disease, have gained prominence [2]. An agreed definition for QoL and patients’ experienced burden of disease is lacking. In this paper, burden of disease is defined as a reflection of the impact of disease, which is suffering due to symptom severity (intensity, frequency, duration), functioning (occupational, social, and leisure activities), and QoL (patients’ satisfaction with health, occupational, social and leisure activities). This indicates that QoL is an acknowledged and thus integrated part of burden of disease [3]. The full scope of burden of disease is rarely assessed in questionnaires. However, numerous instruments have been developed to assess patients’ QoL [2]. These instruments might be used in clinical practice, and are either generic or disease-specific. Generic instruments have the ability to measure the overall QoL. This is especially relevant for people with multimorbidity, where approaches focussing on a single condition are not convenient [4]. In contrast, disease-specific instruments are more able to detect specific symptoms and disease-related changes over time, at least for that specific condition [5]. Although several disease-specific instruments can be used in case of multimorbidity, it might be inappropriate and difficult to attribute a specific complaint, such as fatigue, to one disease. Besides, just completing one or more questionnaire(s) does not in itself improve patient-centred care and shared decision making. To make a questionnaire more relevant in daily healthcare, the outcomes should be integrated in the conversation between the patient and the healthcare provider, such as through visualisation of the results. A need for an instrument that combines the benefits of generic and disease-specific instruments is reinforced. Additionally, this instrument should measure and visualise the burden of one or more chronic conditions, and give appropriate treatment advice. Therefore, the Assessment of Burden of COPD (ABC)-tool will be reformed into the Assessment of Burden of Chronic Conditions (ABCC)-tool for multiple chronic conditions. The current study focusses on COPD, asthma, and/or DM2, because these are common diseases in general practice, and due to funding possibilities.

**Fig. 1:**
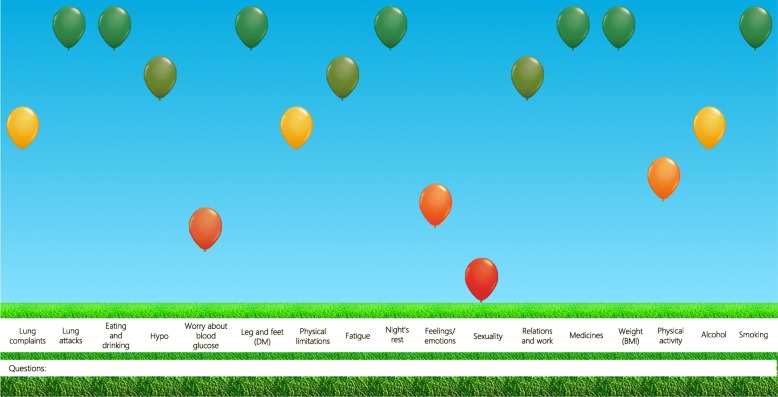
Visualisation of the integrated health status of a person with COPD and DM2

**Fig. 2:**
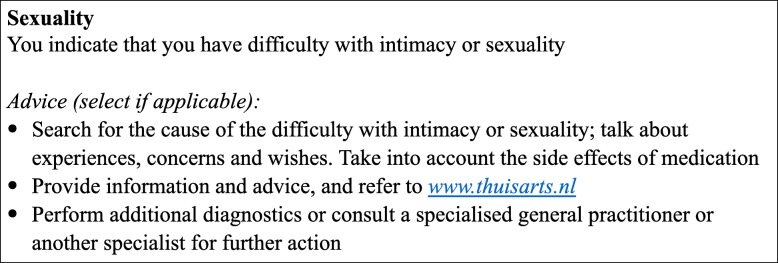
Example of treatment advice (translated from the originally Dutch version)

The ABC-tool was developed in 2014 [6]. The tool measures a patient’s integrated health status. In the current paper, integrated health status is defined as the experienced burden of disease and essential risk factors for the chronic condition(s). The results are visualised and integrated into the conversation between healthcare provider and patient. This conversation is based on the principles of shared decision making. The tool consists of several components, namely the ABC-scale that measures the experienced burden of COPD, objective parameters and risk factors; a visualisation of the outcomes based on cut-off points; and treatment advice [6]. Outcomes of the ABC-scale are visualised using balloons, such as displayed in Fig. 1 for the ABCC-tool. A balloon represents a domain of the burden of disease or a risk factor, and the colour and height indicate a patient’s score on that domain. A red balloon indicates a low score, an orange balloon indicates a moderate score, and a green balloon indicates a high score. The height of the balloons is based on cut-off points. Grey balloons visualise domain scores of the previous visit, which enables to monitor and visualise changes over time. If a patient and healthcare provider select a balloon by clicking on it, treatment advice is shown. Treatment advice include among others self-management advice, suggestions for different treatments, and possibilities for further discussion. The advice is generic and based on current guidelines. An example of advice in the ABCC-tool is shown in Fig. 2. Based on the discussion following the treatment advice, personal care plans can be determined and - where applicable - treatment advice can be operationalised [6]. The ABC-tool has shown to be valid, reliable and effective in improving QoL and perceived quality of care [7, 8]. In general, patients and healthcare providers respond positively to the tool [9]. Based on these positive outcomes, the ABC-tool is currently being implemented in daily primary care in the Netherlands.

The aim of the current study is to assess how the ABC-tool can be transformed into the ABCC-tool for patients with COPD, asthma and/or DM2 aged 18 years and older. Secondary research questions are: 1) does an appropriate questionnaire exist that assesses the burden of asthma or DM2? and 2) which domains should be included in a questionnaire to assess the burden of COPD, asthma, and DM2 in adult patients?

## Methods

The study includes a literature study and a qualitative study, and consists of six consecutive phases. The study was conducted in the Netherlands. In the first phase, requirements were formulated that a questionnaire for the burden of asthma and DM2 should meet. These requirements were based on the requirements that were established during the development of the ABC-scale, and were adapted if needed based on consensus in the research group [6]. The research group consisted of four researchers, including a professor in primary health care (OS), an epidemiologist (EB), a health scientist (AG), and a medical doctor (DC). Secondly, a literature study was conducted to determine whether a questionnaire existed that assesses the burden of asthma or DM2, and that met the predefined requirements. This questionnaire could then serve as a basis for the development of the ABCC-tool. The ABC-scale was based on the validated Clinical COPD Questionnaire [6]. The terms that were included in the searches for asthma and DM2 are displayed in Additional file 1.1. The searches were conducted in October 2018 for DM2 and in November 2018 for asthma. One researcher (DC) assessed the titles and abstracts of the studies regarding asthma, and one researcher (EB) assessed the studies regarding DM2, to extract all existing questionnaires. Other questionnaires were searched via the snowball method. Two researchers (EB and DC) independently assessed whether the questionnaires met the requirements that were formulated in phase 1. The outcomes were cross-checked between the researchers and any disagreement was being resolved through consensus. In the third phase, a first version of the ABCC-scale was established based on the literature study, clinical experience, expert knowledge and the Dutch College of General Practitioners’ guidelines. Fourthly, interviews were conducted with patients and healthcare providers. Patients were recruited through general practices in the Netherlands. Healthcare providers were recruited via e-mail and included general practitioners, internists, pulmonologists, and general practice-based nurses. Interviews with healthcare providers were done face-to-face by one of the researchers (EB or DC). From a constructivism point of view, patients’ experienced burden and healthcare providers’ experiences concerning the experienced burden of patients were gathered. By means of phenomenology, these lived experiences are combined into a synopsis that is practical for daily healthcare practice. The aim of the interviews with healthcare providers was to assess whether the questionnaire and the associated domains were appropriate and sufficient to determine the burden of disease. During the interview, the preliminary ABCC-scale was shown to the healthcare provider. The interview guide consisted of the following topics: 1) do the domains cover the burden of patients with COPD, asthma and DM2?, 2) can the questions be used to identify if there is a problem in the domains of the ABCC-scale?, and 3) are the questions logical and understandable? Patients were interviewed alone, in a duo-interview or in a focus group by one or both of the researchers (EB and/or DC). The aim of the interviews with patients was to determine the experienced burden of disease, and to assess if the questions were logical and understandable. Patients were asked to write down their experienced burden of disease. Subsequently, the topics that were written down were discussed. If no other topics emerged during the interviews, the preliminary ABCC-scale was shown to the participating patients. Patients were asked to complete the questionnaire, and indicate if the questions were logical and understandable. Interviews were conducted until theoretical data saturation, defined as the point at which no new themes emerged from the interviews. The interviews were audio-recorded and transcribed verbatim et literatim. The analyses were performed manually. The transcripts were coded, and emerging themes were identified by two researchers (EB and DC). The themes were compared and interpreted by these researchers. In case of any dispute a third researcher (AS) was consulted in order to take a final decision. The study had an iterative character, i.e. the questionnaire was adapted after several interviews, and was then used in the next interviews. In the fifth phase, the final content of the questionnaire was determined during three meetings in collaboration with an expert group, including among others general practitioners, diabetes mellitus specialists, and pulmonology specialists. Two researchers (EB and DC) made an updated version of the scale before each meeting based on the data from the qualitative interviews and the expert group. During the meeting, all attendees discussed which domains should be included, which questions should address the domains, and whether the questionnaire was comprehensible for patients. Consensus was reached based on clinical expertise, expert knowledge, data from the interviews, and guidelines. Also the length of the questionnaire and balloon chart was taken into consideration. Lastly, cut-off points and treatment advice were determined in an expert group consisting of the research group, one pulmonologist (JV), one general practitioner (PS), and one internist (HB) during two meetings. Two researchers (EB and DC) made a first version of the cut-off points and treatment advice before the meeting, and all content was discussed during the meeting until consensus was reached. Cut-off points and treatment advice were based on the Dutch College of General Practitioners’ guidelines, and formulated by the expert group. These cut-off points determine the height and consequently the colour of the balloon. Each balloon is accompanied by different treatment advice, depending on the cut-off points.

## Results

In the first phase, several requirements for the ABCC-scale were determined, based on the requirements formulated for the ABC-scale [6]. Some requirements were not apposite for the ABCC-tool, such as the possibility to connect with generic QoL instruments, and were therefore not taken into account. Requirements were also added, such as the ability to measure burden of medication use. It was concluded that the questionnaire should: 1) include indicators that provide insight into impairments, disabilities, complaints, and QoL due to COPD, asthma or DM2, 2) measure symptoms, emotions, limitations, social experiences, and burden of medication use, 3) be based on patient input, 4) be easy for both patients and caregivers to manage (takes less than 10 min to complete, includes subscores and a total score, and potential to be self-administered by the patients), 5) be aimed to be used in daily healthcare practice, and 6) have good psychometric properties (validity, reliability, responsiveness).

Secondly, the literature searches were conducted, which resulted in 4820 and 3280 studies for DM2 and asthma, respectively. Seventeen questionnaires were identified for DM2 and 29 questionnaires were identified for asthma. Additional files 1.2 and 1.3 show whether the questionnaires met the requirements. In some cases, it was doubtful if the questionnaire met the requirements, or no information was found in the literature. No instruments were found that met all requirements. Moreover, it was concluded that the use of validated disease-specific questionnaires in the ABCC-scale is not a favourable option, since these questionnaires are not meant to be used in a scale for multiple chronic conditions. In other words, the validated questionnaires would have to be split up in generic and disease-specific questions, losing their validity as a result. Consequently, questions from different questionnaires should be reformulated to make consistencies in the generic questionnaire. Therefore, no existing questionnaires for DM2 or asthma were used as a basis for the ABCC-scale.

During the third phase, a first version of the ABCC-scale was developed. To construct a questionnaire that is applicable both for patients with a single condition and multiple conditions, the scale includes a generic questionnaire and disease-specific questionnaire(s). This furthermore allows for the development of disease-specific questionnaires for other common chronic conditions within the same ABCC-tool. The disease-specific questionnaire(s) will be combined with the generic questionnaire, and they should not be used separately. The patient will receive an individualised single scale, which includes the generic questionnaire, and one or multiple disease-specific questionnaire(s). The outcomes will be visualised in one individualised balloon chart.

After the development of a first version of the ABCC-scale, interviews were conducted with patients and healthcare providers as part of phase four. Eighteen healthcare providers were interviewed, including three general practice-based nurses, three general practitioners, four pulmonologists, six internists, one diabetes nurse, and one pulmonary nurse. Four healthcare providers were male, and fourteen healthcare providers were female. Furthermore, twenty-one patients were interviewed, including six patients with asthma, fourteen patients with DM2, and two patients with COPD. One patient was diagnosed with asthma and DM2. Ten patients were male, and eleven patients were female. The themes that emerged during the interviews are broadly reflected in the domains of the ABCC-tool. Some topics emerged during interviews with healthcare providers but not with patients, such as hypo-unawareness.

In the fifth phase, decisions on the final content of the questionnaires were made based on feasibility, i.e. length of the questionnaire and the balloon visualization, as well as consensus in the expert group. Seven generic domains that determine disease burden were identified, including: 1) physical limitations, 2) fatigue, 3) night’s rest, 4) feelings/emotions, 5) sexuality, 6) relations and work, and 7) medicines. To measure integrated health status, other essential disease parameters and risk factors were added, including 1) weight/body mass index, 2) physical activity, 3) alcohol, and 4) smoking. For the DM2-scale, four additional domains were identified, including: 1) hypoglycaemia (described as hypo), 2) worry about blood glucose, 3) leg- and feet complaints, and 4) eating and drinking. The COPD-scale includes two additional domains: 1) lung complaints, and 2) lung attacks (exacerbations). For the asthma-scale, three additional domains were identified, including 1) asthma complaints, 2) nasal complaints, and 3) lung attacks (exacerbations). Figure 1 displays an example of the balloon visualisation for COPD and DM2. The number of questions per domain range from one to four. Outcomes are scored using a 7-point Likert scale. An open-ended question was added to give a patient the option to address other topics or questions. The scale consists of a total of 21, 23 and 24 questions, for DM2, COPD and asthma respectively.

Cut-off points and treatment advice were determined by the expert group during the last phase. For example, if a patient has an average score of three in the domain feelings/emotions, a red balloon on a height of 30% will be shown, with accompanying treatment advice. The cut-off points are either based on an average score, or are based on specific score combinations, based on consensus in the expert group. An example of treatment advice is shown in Fig. 2 and in Additional file 2.

## Discussion

In this study, the ABC-tool was reformed into a disease-specific ABCC-tool for COPD, asthma, and DM2. The ABCC-scale fulfils the requirement to measure symptoms, emotions, limitations, social experiences and burden of medication use. The scale is based on input from patients, health care providers, and experts, and is aimed at easy administration.

PROMs are widely used to assess patient perspectives on healthcare outcomes. In 2012, the International Consortium for Health Outcomes Measurement (ICHOM) was established to identify a standard set of outcomes that reflects what matters most to patients [10]. Although the potential role of ICHOM within the ABCC-scale was studied, unfortunately no ICHOM set exists for COPD or asthma to this day. Regarding DM2, the ICHOM recommends to routinely assess psychological well-being, diabetes distress and depression [11]. Although these concepts are included in the ABCC-scale, it was not desirable to include the associated questionnaires (the WHO-5, PAID and PHQ-9 respectively) because this would increase the length of the tool, and therefore decrease the feasibility of the tool in daily practice. PROMs either measure general health or disease-specific health, with in most PROMs emphasis on the latter [12]. However, several studies have underlined the relevance of combining the advantages of generic and disease-specific questionnaires [2, 5, 13]. The ABCC-tool may combine the conveniences of both, and as such - and as far as we know - is unique in its kind. Complaints that can originate from several chronic conditions do not need to be attributable to one specific condition. Moreover, patients only need to complete one questionnaire instead of one questionnaire for each condition, and therefore user-friendliness is pursued.

Certainly in case of multimorbidity, care should be person-centred instead of disease-centred, mainly focused on QoL, and promote self-management using agreed personalised goals [4]. The ABCC-tool fits with the vision that care for chronic conditions should not be based on clinical outcomes alone, but also on physical, mental and social well-being [13, 14]. Moreover, it is in line with the thought of integrated care, viewing the patient in a holistic perspective and leading to advice that is tailored to the patient’s needs [15]. Mounting evidence indicates that patient-centered care could be the next step in improving care for people with chronic conditions [16–18]. Furthermore, the ABCC-tool is meant to support self-management, because it helps formulating personal care plans based on the discussion following the treatment advice. The goals in the personal care plan are chosen with the patient in the lead, which might increase the motivation to work on the personal goals in the home setting. This helps patients with a chronic condition to live with the best possible QoL [19]. With regard to diabetes, recommended care has shifted over the past decade to an approach in which individualised patient care and self-management support are essential components [20]. Several studies highlight the importance of communication between patients and healthcare providers in diabetes self-management [21–23]. Health behaviour changes more likely occur if patients actively participate in setting their diabetes self-care agenda [21]. Concerning asthma, self-management education has been shown to reduce urgent healthcare, work or school absences and sleep deprivation [24]. Regarding COPD, several domains such as severity, activity and impact, should be taken into account in order to properly capture the complexity and to provide the best possible patient-centred care [25]. Multifactorial COPD management might significantly improve health-related QoL [26]. Several studies have shown that self-management strategies improve a variety of health-related outcomes for patients with COPD [27, 28].

A strength of the study is that it is based on the principle and development of the ABC-tool, which has shown to improve QoL and experienced quality of care [8]. Besides, the development of the questionnaire was based on a broad input, including a literature study, guidelines from the Dutch College of General Practitioners, and the input of healthcare providers, patients, and researchers. Moreover, a strength of the tool is that it not only quantifies, but also visualises the burden of disease, provides domain-specific practical recommendations, and integrates burden of disease into a conversation based on shared decision making. This might aid the translation of a score on a questionnaire to a tailor-made care plan, and most likely increase the feasibility of the tool in daily healthcare.

Although we have tried to include a diverse group of patients, we cannot ensure to have an adequate representation of the population with COPD, asthma and DM2 (and any combination of these conditions) and their healthcare providers. For example, only one patient with multimorbidity was included in the qualitative study. We aim to increase the number of patients with multimorbidity during the next steps of this research. Furthermore, we did not generate an item-bank and perform item-reduction by means of statistical analysis. Instead, we determined the most important and relevant items based on clinical expertise in expert group meetings.

Further research is imperative to assess the validity and reliability of the ABCC-scale, the (cost)-effectiveness, and the feasibility of the tool. Moreover, research should be conducted to understand the process of implementing the tool in daily practice. It is recommended to update treatment advice once important changes in the guidelines have been made. The ABCC-tool has currently been developed for COPD, asthma, and DM2. The focus on these conditions was chosen because these are common diseases in general practice, and because of funding possibilities. Future aims are to further develop the ABCC-tool for other common chronic conditions, including mental diseases, cancer, and cardiovascular diseases such as heart failure and atrial fibrillation. In recent years, a plea for a new approach has been made, in which treatable traits in airway diseases should be identified and adequately treated [29, 30]. In future research, it is aimed to assess whether the ABCC-tool can play a role in tailored treatments, and whether the tool is able to identify treatable traits.

## Conclusions

This paper describes the development of the ABCC-scale to assess the burden of COPD, asthma and/or DM2, as well as the integrated ABCC-tool. The tool consists of a questionnaire, a visualisation using balloons that is based on cut-off points, and treatment advice. The scale consists of a generic questionnaire, with items that might be relevant for everyone with a chronic condition, as well as disease-specific questionnaires. The generic questionnaire will be combined with any amount of disease-specific questionnaires (up to now: COPD, asthma, and DM2) to form a single personalised scale and balloon chart for each individual patient. The ABCC-tool is intended to be used in daily healthcare practice, is designed to monitor a patient’s integrated health status over time, to facilitate shared decision making, and to stimulate self-management.

## Supplementary information

**Additional file 1.** Health-related quality of life instruments in relation to requirements for a burden of disease instrument for asthma and DM2

**Additional file 2.** Example of treatment advice

## Data Availability

The datasets used and/or analysed during the current study are available from the corresponding author on reasonable request. The ABCC-questionnaire can be requested from the corresponding author.

## Abbreviations

ABCC-tool: Assessment of Burden of Chronic Conditions-tool
ABC-tool: Assessment of Burden of COPD-tool
COPD: Chronic Obstructive Pulmonary Disease
DM2: Diabetes Mellitus type 2
PROM: Patient Reported Outcome Measure
QoL: Quality of Life
