# Supplementary material for: ABC-tool reinvented: development of a disease-specific ‘Assessment of Burden of Chronic Conditions (ABCC)-tool’ for multiple chronic conditions
Source: BMC Fam Pract. 2020 Jan 13;21:11. doi: 10.1186/s12875-019-1075-8 (PMC6958572; doi:10.1186/s12875-019-1075-8)
Supplement: Supplementary file 1 — Additional file 1. Health-related quality of life instruments in relation to requirements for a burden of disease instrument for asthma and DM2 [file 12875_2019_1075_MOESM1_ESM.pdf]

Additional file 1: Health-related quality of life instruments in relation to requirements for a burden of disease instrument for asthma and DM2

1.1: Search terms for asthma and DM2 respectively

((((((((("Patient Reported Outcome Measures"[Mesh]) OR Patient Reported Outcome Measure[Title/Abstract]) OR PROM[Title/Abstract])) OR (("Global Burden of Disease"[Mesh]) OR Burden of Disease[Title/Abstract])) OR ((("Quality of Life"[Mesh]) OR Quality of Life[Title/Abstract]) OR QoL[Title/Abstract])) OR (("Disease Management"[Mesh]) OR Disease Management[Title/Abstract]))) AND ((("Surveys and Questionnaires"[Mesh])) OR questionnaire[Title/Abstract])) AND (("Asthma"[Mesh]) OR asthma[Title/Abstract])

((((((((("Patient Reported Outcome Measures"[Mesh]) OR Patient Reported Outcome Measure[Title/Abstract]) OR PROM[Title/Abstract])) OR (("Global Burden of Disease"[Mesh]) OR Burden of Disease[Title/Abstract])) OR ((("Quality of Life"[Mesh]) OR Quality of Life[Title/Abstract]) OR QoL[Title/Abstract])) OR (("Disease Management"[Mesh]) OR Disease Management[Title/Abstract]))) AND ((("Surveys and Questionnaires"[Mesh])) OR questionnaire[Title/Abstract])) AND (("Diabetes Mellitus"[Mesh]) OR Diabetes[Title/Abstract] OR Diabetes Mellitus[Title/Abstract] OR DM[Title/Abstract] OR DM2[Title/Abstract])

Table S1.2: Properties of Asthma questionnaires measuring quality of life

✓ Tool meets requirements ✗ Tool does not meet requirements ~ Doubtful[a] ? Information not found in the literature

Requirements for a burden of disease instrument

Tool	Symptoms	Emotions	Limitations	Social experience	Treatment	Patient input	Completion time ≤10 min.	Self-administered	Sub + total score	Clinical usability	Validity	Reliability	Responsiveness
ABP[1]	✗	✓	✓	✓	✗	✓	✓	✓	✓	✓	✓	✓	✓
ACCI[2]	✓	✗	✓	✗	✓	✓	✓	✓	✓	✓	✓	~	?
ACQ[3]	✓	✗	✓	✗	✓	✗	✓	✓	✓	✓	✓	✓	✓
ACT[4]	✓	✗	✓	✗	✓	✗	✓	✓	✗	✓	✓	✓	✓
A-IQOLS[5]	✗	~	✓	✓	✗	~	✓	✓	✗	~	✓	~	✓
AIS-6[6]	✗	✓	✓	✓	✗	~	✓	✓	✗	?	~	✓	?
AQ20[7]	✓	✓	✓	✓	✓	?	✓	✓	✗	~	✓	✓	✓
AQL-5D[8]	✗	✓	✗	✓	✓	✗	✓	✓	✗	✗	~	~	?
AQLQ(S)[9]	✓	✓	✓	✗	✗	✓	~	✓	✓	✓	✓	✓	✓
AQLQ-S[10]	✓	✓	✓	✓	✗	✓	✓	✓	✓	✓	✓	~	✓
ASC[11]	✓	✓	✗	✗	✗	✓	?	✓	✓	✓	?	~	?
ASD[12]	✓	✗	✓	✗	✗	✓	?	✓	✗	✗	✓	✓	✓
ASF[13]	✓	✓	✓	✗	✗	~	✓	✓	✓	✓	✓	✓	✓
ASUI[14]	✓	✗	✗	✗	✓	✓	?	✗	✗	✗	✓	✓	✓
ATAQ[15]	✓	✗	✓	✗	✓	✗	✓	✓	✗	✓	✓	?	✓
CARAT(10)[16]	✓	✗	✗	✗	✓	✓	✓	✓	✓	✓	✓	✓	✓
CRQ-SR[17]	✓	✓	✗	✗	✗	✓	✗	✓	✓	✓	✓	✓	✓
LASS[18]	✓	✗	✗	✗	✗	✗	?	✓	✗	✓	✓	~	✓
LWAQ[19]	~	✓	✓	✓	✓	✓	✗	✓	✓	✗	~	✓	~
MAQLQ-Marks[20]	✓	✓	✓	✓	✓	~	✓	✓	✓	✓	✓	✓	✓
Mini-AQLQ[21]	✓	✓	✓	✗	✗	✓	✓	✓	✓	✓	✓	✓	✓
Qol-RIQ[22]	✓	✓	✓	✓	✓	~	?	✓	✗	?	✓	✓	✓
RAND-ACM[23]	✓	✗	✓	✗	✓	✗	?	✓	✗	✓	✓	~	?
RAND-IAQL-12[24]	✗	✓	✓	✓	✓	✓	?	✓	✗	✓	✓	✓	?
RCP3Q[25]	✓	✗	✓	✗	✗	~	✓	✓	✗	✓	?	?	?
Rhinasthma	✓	✗	✓	✗	✓	✓	✓	✓	?	✓	✓	✓	✓
RIQ-MON10[26]	✓	✓	✓	✓	✗	~	✓	✓	✓	✓	✓	✓	~
SAQ[27]	✗	✓	✓	✓	✓	✓	?	✓	✓	~	✓	✓	?
SGRQ[28]	✓	✗	✓	✓	✓	✗	✗	✓	✓	✗	✓	✓	✓

[1] Asthma Bother Profile [2] Asthma Control and Communication Instrument [3] Asthma Control Questionnaire [4] Asthma Control Test [5] Asthma Impact on Quality of Life Scale [6] Asthma Impact Survey 6 [7] Airways Questionnaire-20 [8] Asthma Quality of Life utility index - 5 Dimensions [9] Asthma Quality of Life Questionnaire (Standardized) [10] Asthma Quality of Life Questionnaire-Sydney [11] Asthma Symptom Checklist [12] Asthma Symptom Diary [13] Asthma Short Form [14] Asthma Symptom Utility Index [15] Asthma Therapy Assessment Questionnaire [16] Control of Allergic Rhinitis and Asthma Test [17] Chronic Respiratory Questionnaire-Self Reported [18] Lara Asthma Symptom Scale [19] Living With Asthma Questionnaire [20] Modified Asthma Quality of Life Questionnaire-Marks [21] Mini-Asthma Quality of Life Questionnaire [22] Quality of Life for Respiratory Illness Questionnaire [23] RAND Asthma Control Measure [24] RAND Negative Impact of Asthma on Quality of Life-12 [25] Royal College of Physicians 3-Questions [26] Respiratory Illness Questionnaire Monitoring-10 [27] Severe Asthma Questionnaire [28] Saint George Respiratory Questionnaire

[a] reliability is doubtful, among others, if test-retest is not performed

Table S1.3: Properties of DM2 questionnaires measuring quality of life

✓ **Tool meets requirements** ✗ **Tool does not meet requirements** **~ Doubtful**[a] **? Information not found in the literature**

Requirements for a burden of disease instrument

Tool	Symptoms	Emotions	Limitations	Social experiences	Treatment	Patient input	Completion time ≤10 min.	Self-administered	Sub + total score	Clinical usability	Validity	Reliability	Responsiveness
ADDQoL[1]	✗	~	✓	✓	✗	✓	✓	✓	✓	✓	✓	✓	~
ADS[2]	✗	✓	✗	✗	✗	✗	✓	✓	✗	✓	✓	✓	?
D-39[3]	✓	✓	✓	✓	✓	✗	?	✓	~	✗	✓	~	~
DCP[4]	✓	✓	✓	✓	✓	✗	✗	✓	~	~	✓	~	?
DDS[5]	✗	✓	✓	✗	✗	✓	?	✓	✓	✓	✓	~	✓
DHP-18[6]	✗	✓	✓	✗	✗	✓	✓	✓	~	✓	~	~	?
DIMS[7]	✓	✓	✓	✓	✓	✗	✗	✓	✓	✗	~	~	?
DOQ-30[8]	✗	✗	✓	✓	✓	✓	?	✓	~	✓	?	~	?
DQLCTQ[9]	✓	✓	✓	✓	✓	✓	✗	✓	✓	✗	~	✓	✗
DQOL[10]	✓	✓	✓	✓	✓	✓	✗	✓	✓	~	✓	✓	?
DQOL-BCI[11]	✓	✓	✓	✓	✓	✓	✓	✓	✗	✓	~	~	?
DQOL-R[12]	✓	✓	✗	✓	✓	✓	?	✓	✓	✓	?	~	?
DSC-R[13]	✓	~	✗	✗	✗	✗	?	✓	✓	✗	✓	✓	✓
EDBS[14]	✓	✓	✓	✓	✓	✓	✓	?	✓	?	✓	✓	?
MDQ[15]	✗	✓	✓	✓	✓	✓	?	✓	?	✓	✗	~	?
PAID[16]	✗	✓	✗	✗	✗	✓	✓	✓	✗	✓	✓	✓	✓
PAID-5[17]	✗	✓	✗	✗	✗	✓	✓	✓	✗	✓	✓	~	?

[1] Audit of Diabetes-Dependent Quality of Life [2] Appraisal of Diabetes Scale [3] Diabetes-39 [4] Diabetes Care Profile [5] Diabetes Distress Scale [6] Diabetes Health Profile-18 [7] Diabetes Impact Measurement Scale [8] Diabetes Obstacles Questionnaire-30 [9] Diabetes Quality of Life Clinical Trial Questionnaire [10] Diabetes Quality of Life Measure [11] Diabetes Quality of Life – Brief Clinical Inventory [12] Diabetes Quality of Life – Revised [13] Diabetes Symptom Checklist – Revised [14] Elderly Diabetes Burden Scale [15] Multidimensional Diabetes Questionnaire [16] Problem Areas in Diabetes [17] Problem Areas in Diabetes –5

[a] reliability is doubtful, among others, if test-retest is not performed
