# Supplementary material for: ABC-tool reinvented: development of a disease-specific ‘Assessment of Burden of Chronic Conditions (ABCC)-tool’ for multiple chronic conditions
Source: BMC Fam Pract. 2020 Jan 13;21:11. doi: 10.1186/s12875-019-1075-8 (PMC6958572; doi:10.1186/s12875-019-1075-8)
Supplement: Supplementary file 2 — Additional file 2. Example of treatment advice [file 12875_2019_1075_MOESM2_ESM.pdf]

## Additional file 2: Example of treatment advice

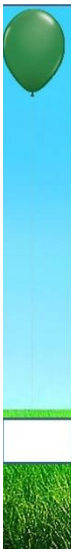

**Sexuality**
You indicate that you have no problems with intimacy or sexuality

*Advice (select if applicable):*

- ☐ None

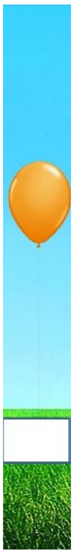

**Sexuality**
You indicate that you have little difficulty with intimacy or sexuality

*Advice (select if applicable):*

- ☐ Search for the cause of the difficulty with intimacy or sexuality; talk about experiences, concerns and wishes. Take into account the side effects of medication
- ☐ Provide information and advice, and refer to *www.thuisarts.nl*

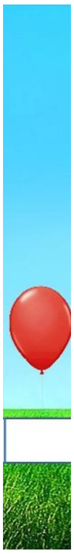

**Sexuality**
You indicate that you have difficulty with intimacy or sexuality

*Advice (select if applicable):*

- ☐ Search for the cause of the difficulty with intimacy or sexuality; talk about experiences, concerns and wishes. Take into account the side effects of medication
- ☐ Provide information and advice, and refer to *www.thuisarts.nl*
- ☐ Perform additional diagnostics or consult a specialised general practitioner or another specialist for further action
